# The *Metschnikowia pulcherrima* Clade as a Model for Assessing Inhibition of *Candida* spp. and the Toxicity of Its Metabolite, Pulcherrimin

**DOI:** 10.3390/molecules28135064

**Published:** 2023-06-28

**Authors:** Dorota Kregiel, Karolina H. Czarnecka-Chrebelska, Hana Schusterová, Renáta Vadkertiová, Adriana Nowak

**Affiliations:** 1 Department of Environmental Biotechnology, Faculty of Biotechnology and Food Sciences, Lodz University of Technology, Wolczanska 171/173, 90-530 Lodz, Poland; adriana.nowak@p.lodz.pl; 2 Culture Collection of Yeasts, Institute of Chemistry, Slovak Academy of Sciences, Dúbravská Cesta 9, 845 38 Bratislava, Slovakia; hana.dudasova@gmail.com (H.S.); renata.vadkertiova@savba.sk (R.V.); 3 Department of Biomedicine and Genetics, Medical University of Lodz, Mazowiecka 5, 92-215 Lodz, Poland; karolina.czarnecka@umed.lodz.pl

**Keywords:** *Metschnikowia pulcherrima*, pulcherrimin, *Candida* spp., cytotoxicity, genotoxicity

## Abstract

Candidiasis is one of the most frequent infections worldwide. In this study, the antimicrobial properties of six strains belonging to the *Metschnikowia pulcherrima* clade were evaluated against twenty *Candida* and *Candida*-related *Filobasidiella neoformans* var. *bacillispora* (syn. *Cryptococcus neoformans*) of different origins, employing the agar cross method. The toxic effect of pulcherrimin, a red metabolite that is responsible for the antimicrobial activities of *Metschnikowia* spp., was evaluated in various experimental models. The results of agar tests showed that the selected *M. pulcherrima* strains inhibited the growth of the *Candida* and non-*Candida* strains. However, inhibition was dependent on the strain and the environment. The presence of peptone, sodium silicate, and a higher incubation temperature decreased the antifungal action of the *M. pulcherrima* strains. Pulcherrimin showed cytotoxic and antiproliferative activity, with oxidative stress in cells leading to apoptosis. More research is needed on the mechanism of action of pulcherrimin on somatic cells.

## 1. Introduction

Invasive infections among immunocompromised patients caused by fungal pathogens have been increasing for over two decades. Most of these life-threatening infections are caused by different *Candida* species in patients in intensive care units. Mortality associated with *Candida* infections ranges from 40% to 60% [1]. Candidemia is probably the most commonly recognized syndrome associated with invasive candidiasis. However, *Candida* spp. can cause many invasive infections, especially in visceral organs, vasculature, bones, joints, eyes, and the central nervous system [2]. Although most systemic infections are caused by *C. albicans*, other species have been reported in clinical trials, including non-*Candida albicans* strains such as *Cyberlindnera jadinii* (syn. *Candida utilis*), *C. tropicalis*, and *C. parapsilosis*. Species representing a potential public health risk include *Pichia kudriavzevii* (syn. *Candida krusei*), *Clavispora lusitaniae* (syn. *Candida lusitaniae*), *Debaryomyces hansenii* (syn. *Candida famata*), and *Torulaspora delbrueckii* (syn. *Candida colliculosa*). These species have biotypes and virulence factors similar to *C. albicans* [3,4].

The last decade has seen the increasing emergence of infections caused by *Candida* species that are resistant to common antifungal agents [1,5,6,7,8,9,10,11,12]. The treatment of microbial infections with antibiotics and fungicidal drugs results in the development of resistance [13]. The resistance of these pathogenic fungi to commonly used antifungal agents greatly contributes to their survival and the rate of successful infections, causing major therapeutic challenges. Consequently, finding new inhibitory agents against these disease-associated fungi is increasingly urgent [14].

Biological control of microbial infections has great potential as an alternative approach to chemical-based methods. One promising method is the use of antagonistic microorganisms to prevent growth and infections. Numerous studies have tried to find effective bioactive agents able to inhibit the growth of *Candida* spp. [15,16,17]. To solve the fungal resistance problem, a further possibility is to investigate the application of yeasts with biocontrol capacity. Different mechanisms, such as competition for nutrients or the secretion of antifungal compounds, have been used to explain their antagonistic activity. Yeast antagonists may produce enzymes that degrade the cell wall or other unknown bioactive agents that could fight against disease-associated *Candida* spp.

*Metschnikowia* Kamienski (1899) is a large ascomycetous genus currently comprising more than 70 species. The *M. pulcherrima* clade contains seven validly described species that share the ability to produce the red pigment pulcherrimin. These yeasts have broad biotechnological potential in various industrial processes, including wine fermentation and low-cost lipid production, as well as as biocontrol agents with strong antimicrobial activity [18]. *Metschnikowia* spp. are mainly isolated from the phyllosphere (flowers, fruits, leaves, and nectars) [19,20]. However, strains belonging to the *Metschnikowia* genus have also been found in gut mycobiota [21].

The *Metschnikowia pulcherrima* clade produces pulcherrimin, which accumulates in environments with Fe ions. Pulcherrimin is visually observed as a red pigment. It is formed when pulcherriminic acid binds iron [22]. Early studies on pulcherrimin show that extracted pulcherrimin is insoluble in all commonly tested organic solvents and acids but can be dissolved in alkali [23]. Therefore, the iron in pulcherrimin is difficult for microorganisms to access.

The antimicrobial activity of *M. pulcherrima* strains mainly depends on iron sequestration [24]. Iron immobilization results in the inhibition of pathogen growth. Therefore, topical application of the *M. pulcherrima* clade and its metabolite pulcherrimin, offers a good alternative for the prevention of a wide spectrum of human infections [25].

The aim of our study was to evaluate the activity of strains belonging to the *M. pulcherrima* clade against *Candida* and *Candida*-related *Filobasidiella neoformans* var. *bacillispora* (syn. *Cryptococcus neoformans*) of different origins. A particular focus was on the potential cytotoxicity and genotoxicity of yeast pulcherrimin, the metabolite responsible for the mechanisms that inhibit the growth of pathogens. We evaluated the induction of oxidative stress and the depletion of mitochondrial membrane potential. We also studied the effects of exposure to pulcherrimin on cell colony formation and adhesion, as well as the morphological changes of cells. To the best knowledge of the authors, this is the first paper presenting the biological activity of pulcherrimin produced by the *M. pulcherrima* clade against different cell lines.

## 2. Results and Disussion

### 2.1. Pulcherrimin Formation

Ten *Metschnikowia* spp. isolates, identified at the Quadram Institute Bioscience (Norwich, UK) on the basis of their barcode sequences (the D1/D2 domains of the LSU rRNA genes), were used [20]. The sequences of the strains (MK612094–MK612103) were deposited in the NCBI database for expert taxonomic verification. However, according to recent research, this molecular method has limited applicability for pulcherrimin-producing *Metschnikowia* yeasts due to ambiguous nucleotides in the amplified D1/D2 domains. Cloning of individual DNA molecules from amplified sequences revealed that the species of the *M. pulcherrima* clade have diverse rDNA repeats in their genomes. A search for ITS sequences in the genome also broadened the range of repeat diversity. The high diversity of ITS and D1/D2 sequences may be inactivated by gradually accumulating mutations. Therefore, as observed by Sipiczki [18,26], finding the relationship of isolates to the type of strain is difficult because a plain blast search can easily lead to false taxonomic inference. The types of strains of the species of the *M. pulcherrima* clade are not separated by clear barcode gaps, making taxonomic identification of pulcherrimin-producing strains difficult or even impossible. Therefore, to quote Lachance, this clade is in “bad need of an expert taxonomic study” [27]. For these reasons, in this study, we refer to the *Mechnikowia pulcherrima* clade with the specification of the tested strains as D1–D10.

**Figure 1:**
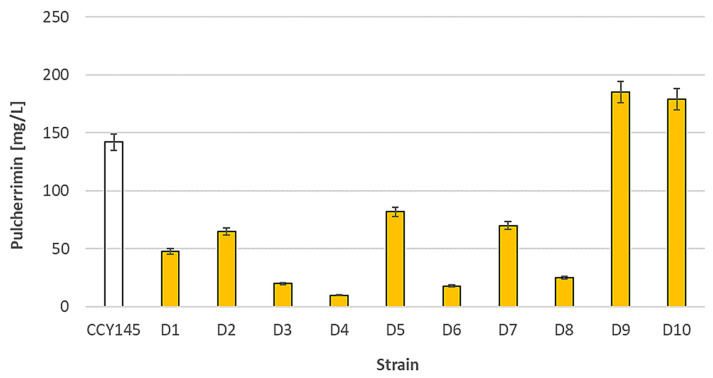
Pulcherrimin formation by strains belonging to the *Metschnikowia pulcherrima* clade in minimal medium with iron ions. Values show the mean ± standard deviation (SD, *n* = 3).

The tested *Metschnikowia* spp. Yeasts were characterized by various levels of pulcherrimin formation. The production of the red metabolite was strain-dependent (Figure 1).

Each tested strain had its own unique pattern of pulcherrimin formation. The most effective producers (strains D9 and D10) were able to form about 180–190 mg/L of pulcherrimin. The D4 strain produced only 10 mg/L. A statistical analysis of the results is presented in Table S1 (Supplementary Materials). We suggest that the formation of pulcherrimin may be dependent on many regulatory mechanisms characteristic for each strain. For example, Lachance suggested that the highest production of red pigment occurs when the iron concentration is slightly inhibitory [27]. Other environmental factors may also interfere with the formation of pulcherrimin, which is a water-insoluble Fe-sequestering chelate.

### 2.2. Inhibition of Candida spp. Strains

**Figure 2:**
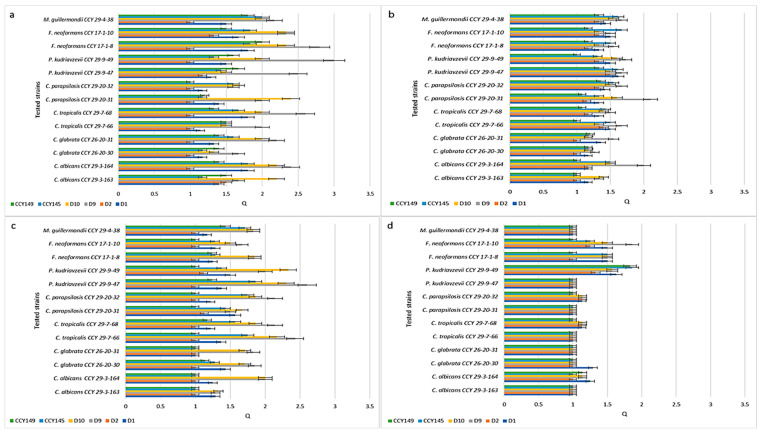
Inhibition of clinical strains by the *M. pulcherrima* clade: (**a**) growth at 22 °C on YED-MB agar; (**b**) growth at 28 °C on YED-MB agar; (**c**) growth at 22 °C on YEPD-MB agar; and (**d**) growth on YEDSi-MB agar.

Figure 2 presents Q values showing the inhibitory activity of the *M. pulcherrima* clade against *Candida* species isolated from humans (clinical strains). To test whether *M. pulcherrima* strains are able to inhibit clinical isolates, four strains of *Metschnikowia* spp. were used from the LOCK Culture Collection (D1, D2, D9, and D10) and two strains from the CCY Collection (CCY145, CCY149). These strains of *Metschnikowia* spp. differed in their production of pulcherrimin (Figure 1 and Table S1—Supplementary Materials). They were screened against 13 *Candida* and *Candida*-related strains using the agar cross method. According to the different inhibition zones obtained, the Q coefficient ranged from 1 (no inhibition) to 3 (very clear inhibition).

In the analysis of the relationship between pulcherrimin production and inhibition activity, for most yeast species there is a positive relationship (Spearman’s Rang correlation), i.e., with the increase in pulcherrimin synthesis, the zone of inhibition increases (Table S2—Supplementary Materials).

The experiments showed that in the control YED-MB agar at temp. 22 °C (Figure 2a), the majority of *M. pulcherrima* yeasts had clear or very clear activity (Q = 1.14 ÷ 3.0) against all the tested clinical strains, except for D2, which showed very weak antifungal activity (from Q = 1.17 for *P. kudriavzevii* CCY 29-9-47 to Q = 1.5 for *C. albicans* CCY 29-3-163). The most active strain was D9, which showed high activity against *P. kudriavzevii* CCY 29-9-49 (Q = 3.0), *F. neoformans* CCY 17-1-8 (Q = 2.8), *C. tropicalis* CCY 29-7-68 (Q = 2.6), and *C. albicans* CCY 29-3-164 (Q = 2.4). However, the antifungal action of *Metschnikowia* strains was dependent on the environment. In agar enriched with peptone YEPD-MB (Figure 2c) and silica YEDSi-MB (Figure 2d), the growth of *Candida* strains was weakly inhibited. The best strain (D9) showed activity against *C. albicans* CCY 29-3-164, expressed as Q, Q = 2.4, Q = 2, and Q = 1.14 in the YED-MB, YEPD-MB, and YEDSi-MB agars, respectively. A similar phenomenon was found after incubation of strains in YED-MB agar at a higher temperature of 28 °C (Figure 2b).

**Figure 3:**
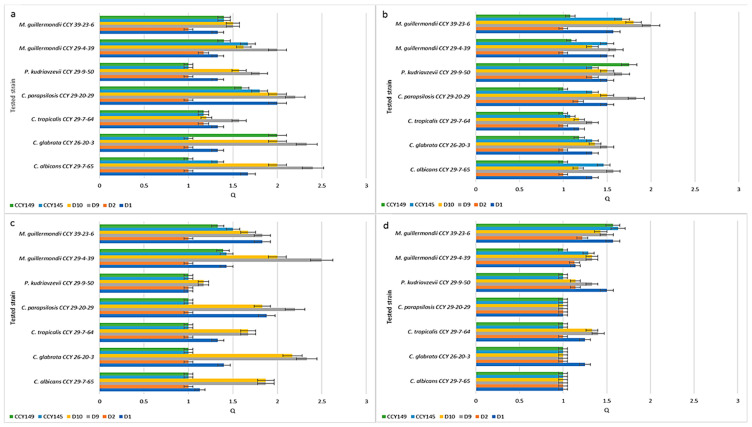
Inhibition of non-clinical strains by the *M. pulcherrima* clade: (**a**) growth at 22 °C on YED-MB agar; (**b**) growth at 28 °C on YED-MB agar; (**c**) growth at 22 °C on YEPD-MB agar; (**d**) growth on YEDSi-MB agar.

**Figure 4:**
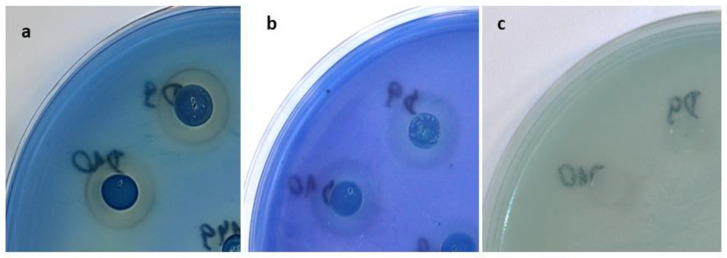
Inhibition zones and pulcherrimin formation (reddish brown rings) by yeast strains D9 and D10 of the *M. pulcherrima* clade: (**a**) growth on YED-MB agar; (**b**) growth on YEPD-MB agar; (**c**) growth on YEDSi-MB agar.

Similar studies were carried out for environmental strains isolated from soil or plants. Figure 3 presents Q values showing the inhibition activity of the *M. pulcherrima* clade against non-clinical *Candida* and related *Candida* species. In this test, 4 yeast isolates from the LOCK Culture Collection (D1, D2, D9, and D10) and 2 strains selected from the CCY Collection (CCY145, CCY149) were challenged against 7 strains from the CCY Collection of different origins. On YED-MB agar at 22 °C (Figure 4a), the majority of *M. pulcherrima* yeasts showed antifungal activity (Q = 1.17 ÷ 2.4) against all tested clinical strains. The D2 strain exhibited weak antifungal activity (Q = 1.17 for *C. tropicalis* CCY 29-7-64 and *M. guillermondii* CCY 29-4-39). The D9 strain showed high activity, especially against *C. albicans* CCY 29-7-65 (Q = 2.4) and *C. glabrata* CCY 26-20-3 (Q = 2.33). It should be noted that the Q value was slightly lower for the environmental strains than for the clinical strains. A similar effect of environmental conditions (the presence of peptone and silica, higher incubation temperature) on the activity reduction of strains from the *M. pulcherrima* clade was found. A statistical analysis of the results is presented in Tables S3 and S4 (Supplementary Materials).

In studies by Robledo-Leal and co-workers [1], *M. pulcherrima* was found to inhibit most isolates of *Candida* spp. *Candida albicans* was the most susceptible species, while *C. tropicalis* showed the least inhibition. We can conclude that both the antifungal activity of *M. pulcherrima* and the sensitivity of *Candida* strains are dependent not only on the species and origin of the strain but also on environmental conditions.

Media composition is known to have a significant influence on the antagonistic activity of microorganisms. Inhibitory effects often depend on specific environmental conditions [28,29]. Higher temperatures stimulate the growth of clinical isolates [30]. This may explain the reduced growth inhibition of clinical *Candida* and related *Candida* species at 28 °C. In turn, an increase in pH level may have a positive effect on the growth of clinical isolates and inhibits the activity of the *M. pulcherrima* clade. This effect was also studied by Türkel and Enerb [25]. They found that when the pH value of the growth medium was increased above 7.0, there was no inhibitory effect of the *M. pulcherrima* strains against the tested microorganisms. It might be that the pigment pulcherrimin does not bind iron at this pH value or that increasing the solubility of pulcherrimin in an alkaline environment also weakens the sequestration effect. Horváth and co-workers [14] suggested that the pH level may influence the appearance of pathogen inhibition.

The production of secondary metabolites often depends on nutritional conditions. This fact may also apply to pulcherrimin, the red metabolite of *M. pulcherrima*. Pawlikowska and co-workers reported that the formation of this metabolite occurs better in a medium low in nutrients [31]. The presence of peptone may favour the growth of clinical strains as well as their proteolytic activity [32]. Iron sequestration through the formation of pulcherrimin can be partially reversed by proteins and peptides. The ability of these compounds to chelate iron is well known [33]. Our results suggest that a decrease in the inhibitory properties of *Metschnikowia* spp. in the presence of peptone may indicate at least partial iron chelation by this supplement.

A more spectacular effect of reducing the antifungal activity of *M. pulcherrima* strains against *Candida* and *Candida*-related species was observed in the agar medium with sodium silicate (YEDSi-MB). Interactions between iron and silica have also been reported [34]. Therefore, in the case of peptides, the interaction between iron and silicate may result in an altered type of iron sequestration: not by reaction with pulcherriminic acid (pulcherrimin formation), but by reaction with peptide particles or silicates. This phenomenon of decreased pulcherrimin formation was observed in the form of changes in the decrease in intensity of the reddish brown colour around the colonies of *Metschnikowia* spp. and decreased inhibition of *Candida* spp. and *Candida*-related strains (Figure 4).

It is interesting to consider the antagonistic activity of strains from the *M. pulcherrima* clade and their pulcherrimin productivity. The D9 strain, which was one of the most effective producers of pulcherrimin, also proved to be the most active against *Candida* and *Candida*-related strains. A similar trend was observed for other good pulcherrimin producers, strains D10 and CCY145 (Figure 1). Strain D2, with the weakest antifungal activity, also turned out to be a poor producer of pulcherrimin. In a study by Türkel and Enerb [25], the tested strains of *M. pulcherrima* produced the same level of red pulcherrimin pigment. However, their antimicrobial activity was different. Hence, their antagonistic effects may depend on other aspects, including extracellular enzymes and the nature of pulcherrimin. Previous studies have shown that pulcherriminic acid, the precursor of red pulcherrimin, may be present in different tautomeric forms in diverse *M. pulcherrima* strains [35]. Therefore, it is possible that the efficacy of iron immobilization by pulcherrimin produced from these strains may be different. Pigmented yeast extract showed some positive results for apoptosis in murine melanoma cell lines and may have positive effects on human health [36]. A similar positive effect on the biological functions of cells was found for pure pulcherrimin obtained from the yeasts belonging to the *M. pulcherrima* clade [37].

To the best of our knowledge, the *M. pulcherrima* clade and its pulcherrimin have not been thoroughly explored for properties relating to candidiasis. Smith and co-workers [38] demonstrated that among the most active yeasts, *Metschnikowia* sp. was capable of protecting human epithelial cells from *Salmonella* spp. invasion. Wilson [39] used *Saccharomyces cerevisiae* as a therapeutic agent against candidiasis. This yeast was found to be a promising candidate for protection against *Candida* infection, as confirmed by the results of in vitro studies. However, in our study, we observed that the antifungal effect also depended on environmental conditions.

**Figure 5:**
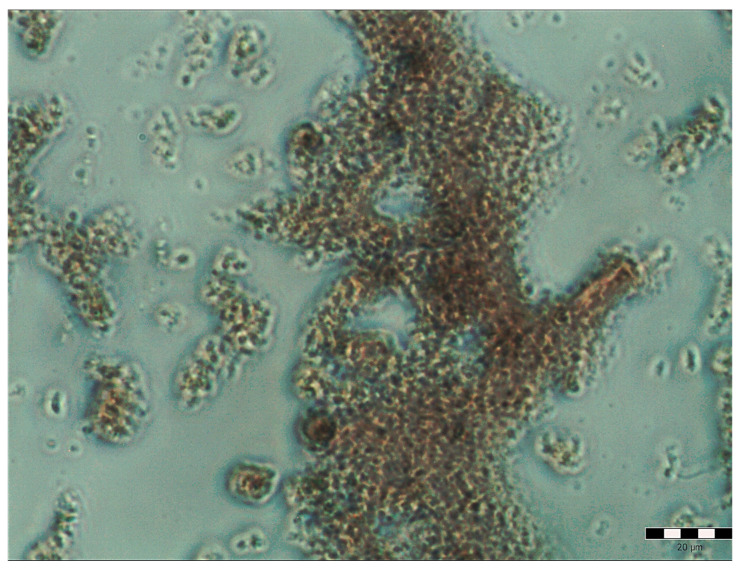
Pure pulcherrimin under a light microscope. The ruler represents 20 μm.

The biological activity of pulcherrimin and its possible cytotoxic/genotoxic effects are relatively understudied. Therefore, we undertook intensive research on the pure pulcherrimin obtained from the *M. pulcherrima* clade (Figure 5).

The purified pulcherrimin obtained was an amorphous red or brownish substance. In water, it formed particles and aggregates of very different sizes, from about 1 μm to over 200 μm.

### 2.3. Cytotoxicity of Pulcherrimin

**Figure 6:**
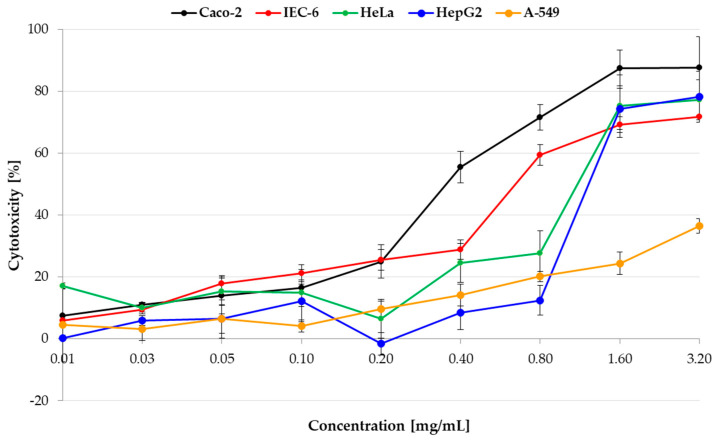
Cytotoxicty of pulcherrimin was determined by the PrestoBlue assay after 48 h exposure. Each data point represents the mean calculated from the fluorescence values of four replicates (±SD).

The first step of the study was to evaluate the cytotoxicity of pulcherrimin in the *PrestoBlue* assay on selected cancer (lung A-549, intestinal Caco-2, cervical HeLa, and liver HepG2) and normal (intestinal IEC-6) cell lines (Figure 6). A statistical analysis of the results is presented in Table S5 (Supplementary Materials). As pulcherrimin is soluble in alkali and not soluble in water solutions (and to avoid the toxic activity of alkali), different concentrations (0.01–3.20 mg/mL) were obtained by suspending the pulcherrimin in an appropriate cell culture medium. For all cell lines, a dose-dependent cellular response was observed. For the three cell lines (A-549, HeLa, and HepG2) up to the tested concentration of 0.20 mg/mL, cytotoxicity was weak, reaching a maximum of 9.61% ± 2.64% (for A-549). It then increased proportionally to a concentration between 0.20 and 3.20 mg/mL (*p* ≤ 0.05). Cytotoxicity was higher (and similar) for intestinal Caco-2 and IEC-6 cells at a concentration of 0.20 mg/mL, at 25.04% ± 5.47% and 25.53% ± 3.34%, respectively (*p* ≤ 0.05). The strongest cytotoxicity was observed for the Caco-2 cells. At the highest concentration of pulcherrimin (3.20 mg/mL), the cytotoxicity was 87.58% ± 10.05% (*p* ≤ 0.05). By comparison, the cytotoxicity at this concentration was 78.21% ± 8.26% for HepG2, 77.20% ± 6.43% for HeLa, 71.82% ± 0.31% for IEC-6, and 36.47% ± 2.35% for A-549 (*p* ≤ 0.05). The cell line most sensitive to pulcherrimin was Caco-2, and the least sensitive was A-549. It can be concluded that, up to a certain concentration, pulcherrimin shows no or weak cytotoxicity against the tested cell lines.

The IC_50_ values (i.e., the concentration of the test compound required to reduce cell viability by 50% relative to the control) were read from the dose-dependence curves (Table 1). They confirmed that pulcherrimin had the most cytotoxic effect on intestinal cells, primarily on cancerous Caco-2, followed by normal IEC-6. A-549 lung cancer cells proved to be the most resistant to pulcherrimin, as the cytotoxicity after 48 h was not high enough to calculate the IC_50_ value.

**Figure 7:**
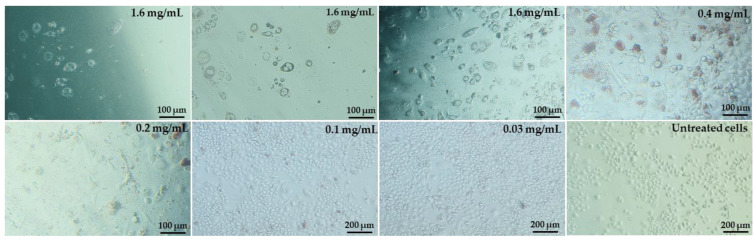
Changes in the monolayer of Caco-2 cells after 48 h exposure to selected concentrations of pulcherrimin (Nikon Ts2 with EMBOSS contrast, Tokyo, Japan), 10× objective.

During the culture of cells with pulcherrimin, we observed changes in the morphology of the cell monolayer (Figure 7). Example microphotographs of selected pulcherrimin concentrations are included for the Caco-2 line, for which pulcherrimin proved to be the most cytotoxic. Cells in the untreated control formed a regular and homogeneous monolayer with well-defined cell membranes and cell nucleuses, as well as clearly visible cytoplasm, including granules. No cell lysis or damage to the monolayer was observed. The cells did not change shape and adhered well to the substrate. After 48 h of exposure to pulcherrimin, the greatest changes in the morphology of the monolayer were observed at the highest concentrations, such as 1.6 mg/mL. The changes included a loss of confluence in the monolayer, a decrease in cell density, and a change to a more rounded or elongated shape. Some cells were partially or fully detached from the substrate compared to the untreated control.

**Figure 8:**
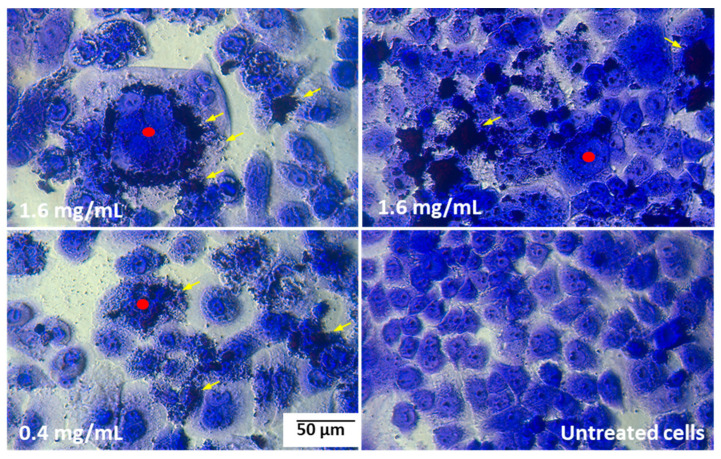
Example microphotographs of Caco-2 cells after 48-h exposure to pulcherrimin (Nikon Ts2 with EMBOSS contrast, Tokyo, Japan) stained with 0.4% crystal violet, 20× objective. Yellow arrows indicate pulcherrimin attached to the cell membranes; red points show swollen cells.

It was found that the pulcherrimin adhered tightly to the cell membranes, as is visible in the microphotographs as darker patches on the cell surfaces and could not even be washed off during the staining procedure. By adhering tightly to the cells, pulcherrimin caused them to swell, and the cell nucleus disintegrated (Figure 8).

Generally, microscopic analyses confirmed the quantitative cytotoxicity results. The damaging activity of pulcherrimin is probably attributable to physical lesions caused by the particles.

The biological activity of pulcherrimin has been demonstrated against a variety of microorganisms, including human pathogens such as the bacteria *Escherichia coli*, *Proteus vulgaris*, and *Staphylococcus aureus*, and the yeasts and fungi *Candida albicans*, *Aspergillus* spp., *Fusarium* spp., *Alternaria* spp., *Aureobasidium* spp., *Saccharomyces* spp., and *Rhodotorula* spp. [18,25,37,40,41]. Pulcherrimin has been found to change the morphology of *Schizosaccharomyces pombe* yeast during cultivation in a culture medium compared to an untreated control, causing abnormal multiple cell divisions [37].

Jayalakshmi et al. [42] demonstrated the cytotoxic activity of pulcherrimin isolated from *Bacillus subtilis* SU-10, which was evaluated by the 3-(4,5-dimethylthiazol-2-yl)-2,5-diphenyltetrazolium bromide (MTT) assay against liver HepG2 and normal fibroblast-like kidney Vero (from African green monkey) cell lines. The IC_50_ value of pulcherrimin for both cell lines was 0.3 mg/mL. This value is similar to the IC_50_ value for Caco-2 cells in the current study. To date, the best known metabolite of another yeast (*Saccharomyces cerevisiae*) with cytotoxic activity against many cell lines (e.g., HeLa, HepG2) is the polysaccharide β-glucan [43,44].

### 2.4. Genotoxicity

**Figure 9:**
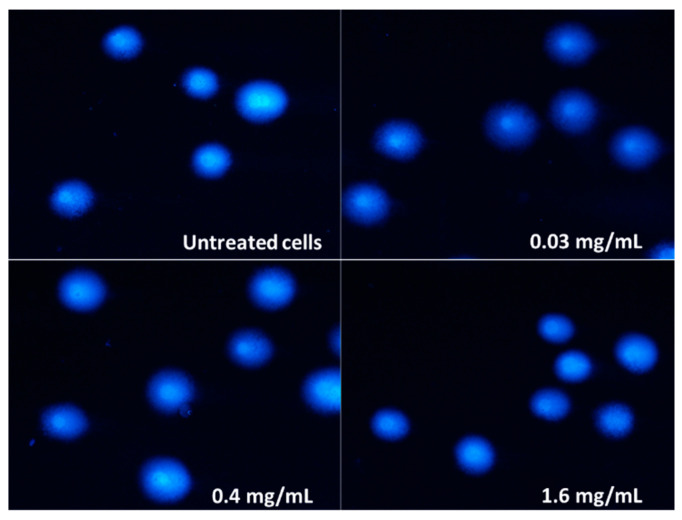
Randomly selected images of DAPI-stained comets exposed to 0.4 mg/mL and 1.6 mg/mL pulcherrimin (Nikon Eclipse Ci H600L, Tokyo, Japan), 20× objective.

Since pulcherrimin showed the strongest cytotoxic activity against Caco-2 intestinal cells, all subsequent studies were continued on this cell line. The genotoxicity was evaluated by the comet assay. The negative control (untreated cells in vehicle) displayed a genotoxicity of 4.31% ± 0.86%, while for the positive control (50 µM H_2_O_2_), the genotoxicity was 88.45% ± 1.82%. As shown in Table 2 and Figure 9, pulcherrimin showed no (≤5.00%) or very weak (≤10.00%) ability to induce DNA damage, regardless of the concentration, and even when the concentration was higher than IC_50_ (i.e., 0.36 mg/mL).

### 2.5. Oxidative Stress Induction and Influence of Pulcherrimin on Mitochondrial Membrane Potential

**Figure 10:**
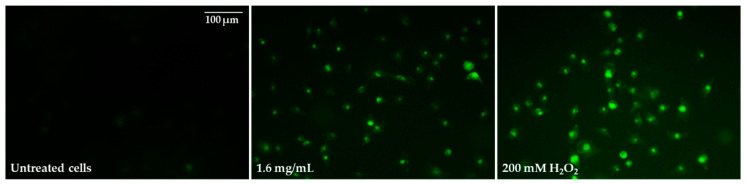
Microphotographs of 2′,7′-dichlorofluorescin diacetate-stained Caco-2 cells after 48-h exposure to 1.6 mg/mL of pulcherrimin. Positive control: 200 mM H_2_O_2_. Fluorescence microscope (Nikon Eclipse Ci H600L, Tokyo, Japan), 20× objective.

In the next step, the ability of selected pulcherrimin concentrations to induce oxidative stress was evaluated. Pulcherrimin-treated Caco-2 cells resulted in a dose-dependent rise in ROS generation (Table 3). As shown in Table 3, concentrations from 0.4 mg/mL and higher caused strong (*p* ≤ 0.05) ROS induction, up to average DCF fluorescence values of 222% ± 3.4%. Intracellular ROS generation was visualized by fluorescence microscopy (Figure 10). In cells exposed to pulcherrimin (1.6 mg/mL), ROS production was increased by mitochondrial impairment, which oxidised 2,7-dichlorofluorescein, which in turn emitted bright fluorescence.

ROS induction was correlated with H_2_O_2_ release, which is one of the ROS (Table 4). At high concentrations, H_2_O_2_ can cause cytotoxicity in cells. Caco-2 cells generated significant H_2_O_2_ (*p* ≤ 0.05) after exposure to pulcherrimin, but not in a dose-dependent manner. The release of H_2_O_2_ for the positive control (10 mM H_2_O_2_) was 21.5 ± 2.9 µM.

**Figure 11:**
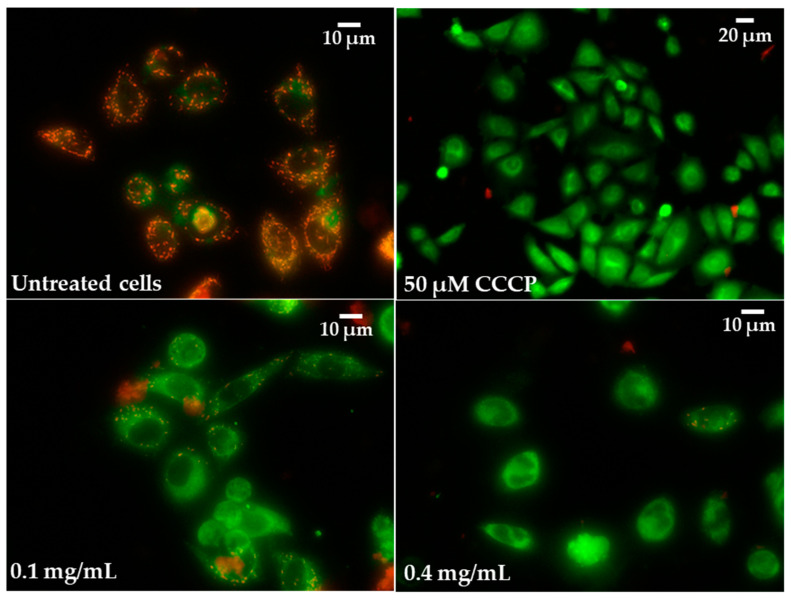
Microphotographs of mitochondrial membrane potential (MMP) in Caco-2 cells were determined with tetraethylbenzimidazolylcarbocyanine iodide (JC-1) as a fluorescent probe staining method after exposure to 0.1 and 0.4 mg/mL of pulcherrimin. CCCP—cyanide m-chlorophenylhydrazone as a positive control. Fluorescence microscope (Nikon Eclipse Ci H600L, Tokyo, Japan), 10× and 20× objectives.

Increased ROS generation and H_2_O_2_ release in Caco-2 cells were associated with reduced MMP. Significant (*p* ≤ 0.05) increases in mitochondrial depolarisation was observed for all tested pulcherrimin concentrations. At a concentration of 1.6 mg/mL, it was 59% ± 1.9%, while for the positive control (50 µM CCCP), it was 43% ± 1.9% (Table 5). Generally, the reduction in MMPs was inversely proportional to pulcherrimin concentration. Figure 11 shows Caco-2 cells with normal (high) MMP, where JC-1 dye forms regular red-orange-coloured aggregates. In the case of reduced MMP, Caco-2 cells emit green fluorescence as JC-1 forms monomers, and aggregates occur much less frequently (Figure 11). This may suggest the early stages of cell apoptosis through the mitochondrial pathway.

There are no studies on ROS induction by pulcherrimin in cell lines. Fernandez-San Millan et al. [45] showed that culture filtrates containing 35 different identified metabolites produced by *M. pulcherrima* induced intracellular ROS levels after DCHF-DA staining in *Botrytis cinerea*. The same metabolites also contributed to the loss of membrane integrity after staining with propidium iodide.

### 2.6. Effect of Pulcherrimin on Colony Formation

**Figure 12:**
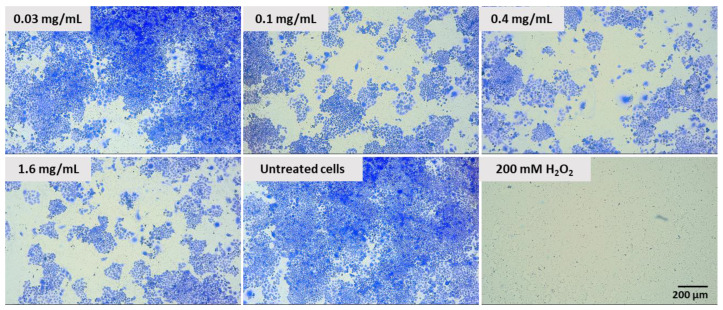
Colonies produced by Caco-2 cells after plating 1000 cells and 7 days of incubation. Cells treated with different concentrations of pulcherrimin for 60 min. Positive control: 200 mM H_2_O_2_. Cells in negative control: not treated. Nikon Ts2 with EMBOSS contrast (Tokyo, Japan), 10× objective.

The proliferative capability of Caco-2 cells treated with pulcherrimin was qualitatively/visually determined based on their ability to form colonies in a colony-forming assay. Pretreatment of Caco-2 cells with different concentrations of pulcherrimin inhibited cell colony formation (Figure 12). The sizes and numbers of colonies were significantly lower after treatment with pulcherrimin, depending on the dose. This confirms the antiproliferative effect of pulcherrimin.

### 2.7. Adhesion Assay

**Figure 13:**
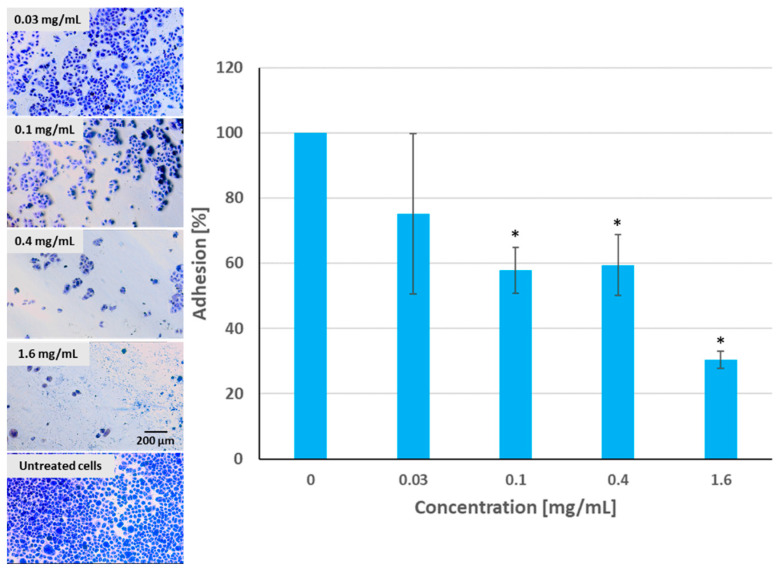
Caco-2 cell adhesion to the substrate after exposure to pulcherrimin measured after staining with 0.4% crystal violet. Each data point represents the mean ± SD (*n* = 4). * Results significantly different from unexposed cells, *p* ≤ 0.05. Random fields photographed, 10× objective (Nikon Ts2 with EMBOSS contrast, Tokyo, Japan).

As can be seen in Figure 12, pulcherrimin inhibited the adhesion of Caco-2 cells to the substrate in a dose-dependent manner (the higher the concentration, the weaker the percentage of cellular adhesion) compared to the untreated control cells. Pulcherrimin at a concentration close to the IC_50_ inhibited cell adhesion by about 40%. A higher cytotoxic concentration (1.6 mg/mL) inhibited adhesion by almost 70%. This trend was confirmed by microphotographs (Figure 13).

Previously, pulcherrimin has been shown to exhibit anti-adhesive and antibiofilm activity against microorganisms such as *Asaia lannensis* and *Bacillus subtilis* bacteria [37,46].

### 2.8. Cells Morphology Assessment after Fluorescent Staining

**Figure 14:**
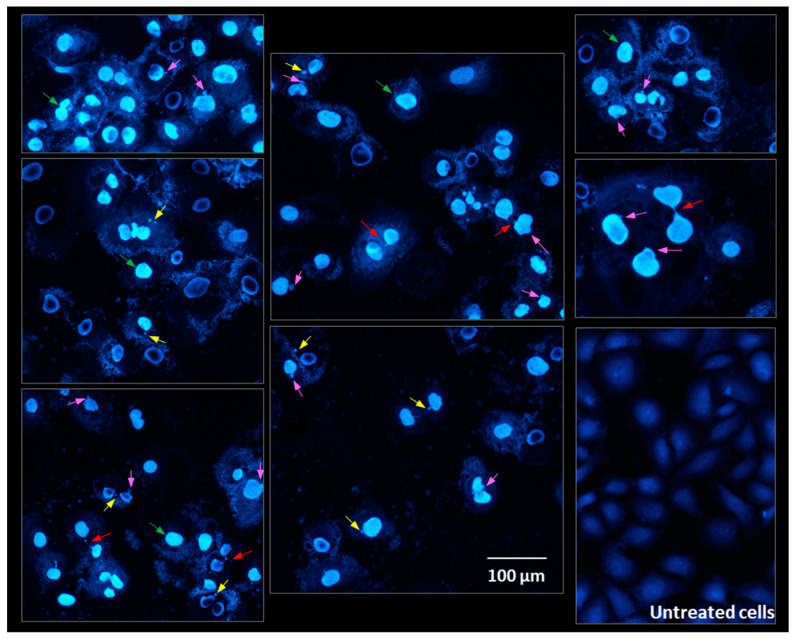
Microphotographs of Caco-2 cells stained with 4′,6-diamidino-2-phenylindole (DAPI) after 24-h exposure to 1.6 mg/mL pulcherrimin observed under a fluorescence microscope (Nikon Eclipse Ci H600L, Tokyo, Japan), 20× objective. Arrows: chromatin condensation (green); nucleoplsmatic bridges (red); micronuclei (yellow); nuclear buds (pink).

Figure 14 shows nuclear and cell morphology after exposure to different concentrations of pulcherrimin and DAPI staining. Untreated Caco-2 cells (intact cells) stained with DAPI were rhomboid in shape. The nuclei were stained homogenously and emitted only light fluorescence. After exposure to pulcherrimin, chromatin condensation was observed in many cells. Nuclear fragmentation and chromatin condensation are considered to be the main symptoms of apoptosis. In addition, characteristic changes were observed in the cell nuclei, such as micronuclei (MN), nucleoplsmatic bridges, and nuclear buds. These abnormalities indicate genotoxicity and changes at the chromosome level. Micronuclei can form as a result of damage to the karyokinetic spindle during mitosis, when whole chromosomes are lost. They can also originate from acentric fragments resulting from chromosomal or chromatid breaks [47]. To be considered micronuclei, they should have an intact nuclear membrane, be located within the same cytoplasm, and have the same pattern and intensity of staining. They may touch but not overlap, they should be distinguishable in the cytoplasm, and the cell membrane must be intact. Furthermore, they may not be connected to the main nucleus. The micronuclei present in our microphotographs appear to meet these requirements. However, induction of MN should be confirmed in the cytokinesis-block micronucleus cytome assay. Nucleoplsmatic bridges can form from dicentric or polycentric chromosomes. The two centromeres of the chromasomes are pulled to opposite poles of the cell, and the chromatin in the resulting bridge is surrounded by a nuclear membrane [47,48]. In some cases, cells may contain structures resembling micronuclei, which are not, because they are not the result of mitosis. This is the result of budding interphase nuclei known as nuclear buds. Buds are connected to the nucleus by a stalk of nucleoplasmic material, depending on the stage of budding [47].

**Figure 15:**
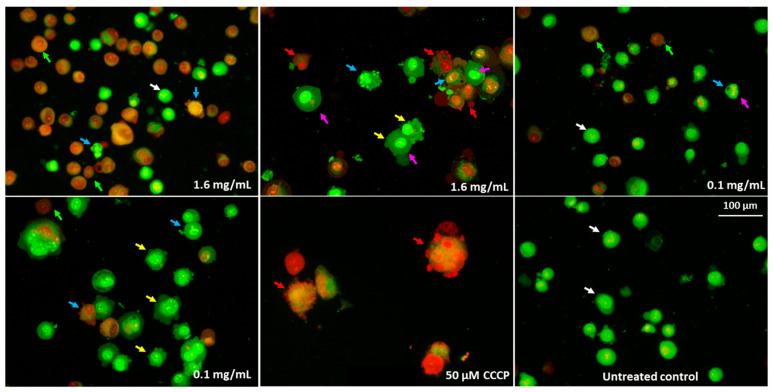
Fluorescence microphotographs of Caco-2 cells double stained with acridine orange/propidium iodide (AO/PI) after 24-h exposure to pulcherrimin (Nikon Eclipse Ci H600L, Tokyo, Japan), 20× objective. Arrows: viable cells (white); membrane blebbing and cell shrinkage (yellow); chromatin condensation (pink); early apoptosis (blue); late apoptosis (red); secondary necrosis (green).

Acridine orange/propidium iodide (AO/PI) staining analyses were also conducted, according to the criteria developed by Baskić et al. [49] and Salim et al. [50] (Figure 15). The AO intercalates in both living and dead cells and generates green fluorescence. In contrast, PI intercalates only into cells with permeable membranes and generates red fluorescence [49]. Green fluorescence with an intact structure of the chromatin was characteristic of the control cells (viable). Orange fluorescence, cell membrane blebbing, condensation, and fragmentation of nuclear material, as well as cell membrane blebbing and cell shrinkage, were symptoms of apoptosis. The bright-red fluorescence of nuclei indicated necrosis. The ability to induce apoptosis by pulcherrimin should be confirmed, for example, by examining the activity of caspases 3/7 and 9.

To the best knowledge of the authors, there are no previous studies in the literature on the activity of pulcherrimin on cell lines. The present study on the biological activity of pulcherrimin against selected cell lines should also be confirmed on other lines.

## 3. Materials and Methods

### 3.1. Yeast Strains

Ten strains of the *M. pulcherrima* clade were isolated from Polish fruits, identified in the NCYC Collection (Norwich, UK), and deposited in the Culture Collection LOCK (Lodz University of Technology, Lodz, Poland). Two collection strains, *M. pulcherrima* CCY145 and CCY149, were also used in the study [20]. As well as *Metschnikowia* spp., twenty strains of different origins (13 of clinical origin and 7 of non-clinical origin) belonging to the genera *Candida* and closely related to this genus were obtained from the Culture Collection of Yeasts CCY (Slovak Academy of Sciences, Bratislava, Slovakia) and tested (Table 6).

The yeast cultures were stored on wort agar slants at 4 °C.

### 3.2. Antifungal Activity

*Candida* strains were grown in 10 mL MEB broth (Merck, Darmstadt, Germany) at 22 °C (non-clinical strains) or 28 °C (clinical strains) for 48 h. The cultures (~10^7^ cfu/mL) were spread on solid methylene blue agar with citrate-phosphate buffer (0.1 M, pH 4.6) with different modifications (Table 7).

When the surface of the microbial plates dried, 4 μL of *Meschnikowia* spp. cultures (~10^7^ cfu/mL, 4 μL) were dropped on each plate in triplicate. The plates were incubated at 22–28 °C for 2 days. After incubation, the diameters of the colonies and the inhibition zones were measured manually and expressed in mm [37]. Similar studies were also performed using methylene blue agar YEDSi-MB with 0.1% SiO_2_ (pH = 6.8). The antifungal activity was determined by the coefficient Q, which was the ratio of the diameter of the inhibition zone to the diameter of the colony.

### 3.3. Chemicals and Other Materials

High-glucose and low-glucose Dulbecco’s Modified Eagle’s Medium (DMEM), RPMI 1640, DMEM:Ham’s F12 (1:1, *v*/*v*), Ham’s F12, phosphate buffer saline (PBS) for cell cultures, 4-(2-hydroxyethyl)-1-piperazineethanesulphonic acid (HEPES), streptomycin-penicillin mixture for cell cultures, insulin, 4′,6-diamidino-2-phenylindole (DAPI), 2′,7′-dichlorofluorescin diacetate (DCFH–DA), trypan blue, paraformaldehyde, hydrogen peroxide (H_2_O_2_), cyanide m-chlorophenylhydrazone (CCCP), a Fluorometric Hydrogen Peroxide Assay Kit (MAK 165), LMP (low melting point), and NMP (normal melting point) agaroses, sodium chloride (NaCl), Triton X-100, EDTA, Tris, and sodium hydroxide (NaOH) were purchased from Merck Life Science, Warsaw, Poland. Foetal bovine serum (FBS), GlutaMAX^TM^, TrypLE^TM^ Express, PrestoBlue, tetraethylbenzimidazolylcarbocyanine iodide (JC-1), and roux flasks T25 and T75 were purchased from Thermo Fisher Scientific, Waltham, MA, USA. In addition, 6- and 96-well plates as well as serological pipettes (Greiner Bio-One GmbH, Kremsmünster, Austria) and 8-well IBIDI LabTek II CC2 chambered coverslips were purchased from Biokom Systems, Janki, Poland.

### 3.4. Obtaining Pure Pulcherrimin

Pulcherrimin was obtained from the *Metschnikowia* sp. LOCK 1144 culture in minimal broth [1% glucose (*w/v*), 0.3% (NH_4_)_2_SO_4_ (*w/v*), 0.1% KH_2_PO_4_ (*w/v*), 0.05% MgSO_4_ × 7H_2_O (*w/v*), 0.05% yeast extract (*w/v*), 0.05% FeCl_3_ (*w/v*)] after 48-h incubation on a rotary shaker (130 rpm) at 25 °C. Pulcherrimin was extracted with methanol (50 mL of 99.8% methanol per 10 g of wet yeast biomass) at 4 °C, then purified by dissolution in NaOH. Precipitation in HCl was repeated three times. Finally, the red pigment was collected by centrifugation and frozen at −20 °C. Quantitative determination of pulcherrimin was conducted spectrophotometrically at the maximum absorption wavelength of 410 nm, according to our previous study [31]. As pulcherrimin is insoluble in water solvents, the stock was prepared by suspending it in a suitable cell culture medium, and the stock concentration was 31.65 mg/mL. The pulcherrimin was stored at −20 °C until analysis.

### 3.5. Cell Cultures

The following adherent cell lines were used in the research: A-549 (human lung alveolar adenocarcinoma), Caco-2 (human colon adenocarcinoma), HeLa (human cervical adenocarcinoma), Hep-G2 (human hepatocellular carcinoma), and IEC-6 (rat normal small intestine). The A-549, Caco-2, and Hep-G2 cell lines were purchased from Cell Line Service GmbH (Eppelheim, Germany). The IEC-6 cells were purchased from DSMZ German Collection of Microorganisms and Cell Cultures GmbH (Germany). The HeLa cells were donated anonymously. Caco-2 and HeLa cells were cultured in high-glucose DMEM, A-549 cells in DMEM:Ham’s F12 (1:1, *v*/*v*), Hep-G2 in Ham’s F12, and IEC-6 in low-glucose DMEM: RPMI 1640 (1:1, *v*/*v*), with the addition of 5% (A-549, Hep-G2, IEC-6) or 10% (Caco-2, HeLa) FBS, 2 mM (A-549, Hep-G2, IEC-6) or 4 mM (Caco-2, HeLa) GlutaMAX^TM^, 25 mM HEPES, a mixture of 100 µg/mL streptomycin/100 IU/mL penicillin, and 0.1 U/mL insulin (IEC-6). All cell lines were incubated at 37 °C in the presence of 5% CO_2_ in a humidified atmosphere for 5–10 days to reach 80% confluence. Two to three times a week, the cells were washed with PBS (pH 7.2), and the medium was replaced with fresh medium. TrypLE^TM^ Express was used to detach the cells from the culture (37 °C, 6–12 min, depending on the cell line). The cells were centrifuged (307× *g*, 5 min), decanted, and re-suspended in a fresh culture medium. The cells were ready to use after counting in a hemacytometer and estimation of cell viability by trypan blue staining (at least 90%).

### 3.6. PrestoBlue Assay

The cytotoxicity of pulcherrimin was evaluated with *PrestoBlue,* a resazurin-based cell viability reagent. A total of 10,000 (Caco-2, HepG2), 5000 (A-549, HeLa), or 20,000 (IEC-6) cells/well were placed in black 96-well plates and incubated (24 h, 37 °C, 5% CO_2_). Next, the medium was removed, and suspensions of pulcherrimin in an appropriate culture growth medium were added. The final tested concentrations of pulcherrimin were (mg/mL): 0.01; 0.03; 0.05; 0.1; 0.2; 0.4; 0.8; 1.6, and 3.2. Unexposed cells were used as a negative control. The samples were exposed to pulcherrimin for 48 h (37 °C, 5% CO_2_). All concentrations and controls were tested in four replicates. Changes in cell morphology were documented only for Caco-2 cells in an inverted microscope (Nikon Ts2 with EMBOSS contrast, Tokyo, Japan, and Jenoptic Subra Full HD Colour digital camera). After the exposure time, the pulcherrimin suspensions were aspirated, and *PrestoBlue* (10% solution in PBS) was added to each well. The samples were incubated for a further 2 h (37 °C, 5% CO_2_). The fluorescence was measured in a microplate reader (TriStar2 LB 942, Berthold Technologies GmbH Co. KG, Bad Wildbad, Germany) at λ_ex_ 560 nm and λ_em_ 590 nm. The fluorescence of untreated cells represented 100% viability. Cell viability (%) was calculated as (sample fluorescence/control fluorescence) × 100%. Cytotoxicity (%) was calculated as 100-cell viability (%). The results were presented as the mean ± standard deviation (SD). The mean error of the method is up to 10%. The IC_50_ values were estimated from the growth curves. Additionally, Caco-2 cells exposed to 0.4 mg/mL pulcherrimin were fixed (3.7% paraformaldehyde, 15 min, ambient temperature), stained (0.1% crystal violet), and observed under an inverted microscope (total magnification 200×).

### 3.7. Single Cell Gel Electrophoresis Assay

Selected concentrations of pulcherrimin (0.0.1, 0.03, 0.1, 0.4, and 1.6 mg/mL) were measured in Eppendorf tubes along with Caco-2 cells in the amount of 1 × 10^5^ cells/sample (the final volume was 1 mL). The negative control contained cells suspended in the culture medium. The cells were exposed for 60 min at 37 °C. The positive control was 50 µM H_2_O_2_ after 10 min exposure on ice. After exposure to pulcherrimin, the cells were centrifuged (15 min, 4 °C, 182× *g*), decanted, and LMP agarose was added at 37 °C. The suspension was spotted on warm NMP double-layered slides and covered with coverslips (hot plate ZF6 Premiere Slide Warmer). The samples were placed on a Chilling Plate for Comet Assay Slides (Cleaver Scientific, Rugby, UK) and allowed to solidify. Alkaline lysis was performed with the buffer (2.5 M NaCl, 1% Triton X-100, 100 mM EDTA, 10 mM Tris, pH 10), and the slides were incubated (60 min, 4 °C). The lysis buffer was decanted, and the slides were flooded with the unwinding buffer (300 mM NaOH, 1 mM EDTA) (20 min, 4 °C). Next, electrophoresis was performed in an electrophoretic buffer (300 mM NaOH, 1 mM EDTA, pH > 13) for 20 min using an electrophoresis apparatus (CSL-COM20, Cleaver Scientific) at a voltage of 21 V and a current of 29 mA. The slides were neutralised in water, allowed to dry, and then stained with 1 µg/mL DAPI (60 min, 4 °C). Comet analysis was performed in the dark under a fluorescence microscope (Nikon Eclipse Ci H600L, Tokyo, Japan) with a total magnification of 200×. The microscope was equipped with a camera (Nikon Digital Sight DS-U3, Tokyo, Japan) and Lucia Comet v.7.0 software (Laboratory Imaging, Prague, Czech Republic). In each trial, 50 randomly selected comets were analysed, based on the parameter determining the percentage of DNA in the comet’s tail. The results were presented as the mean ± S.E.M.

### 3.8. Reactive Oxygen Species (ROS) and Hydrogen Peroxide Generation

For both assays, 10,000 Caco-2 cells were seeded in each well of a 96-well black plate in a complete culture medium. The cells were exposed to selected concentrations of pulcherrimin (0.03, 0.1, 0.4, and 1.6 mg/mL), as described in Section 3.5.

After exposure to pulcherrimin, the pulcherrimin was aspirated, and the cells were washed with PBS. DCFH–DA (20 µM) was added to each well with culture media without FBS, and the samples were incubated for 40 min (37 °C, 5% CO_2_). The untreated (negative) control contained cells in DMEM (without FBS), while the positive control contained cells in DMEM (without FBS) with 200 mM H_2_O_2_. After incubation, the fluorescence was measured (λ_ex_ 490 nm and λ_em_ 530 nm). The average DCF fluorescence was determined as a percentage (%) relative to untreated cells, which was assumed to be 100%. For microscopic observations, Caco-2 cells were cultured in 8-well IBIDI LabTek II CC2 chambered coverslips (50,000 cells/well). The intracellular fluorescence of the cells was observed under the abovementioned fluorescence microscope (Nikon Eclipse Ci H600L, Tokyo, Japan) using a 20× objective and imaging software (NIS-elements BR 3.0, Nikon, Tokyo, Japan). The increased intensity of intracellular fluorescence was indicative of an increased generation of ROS. A Fluorometric Hydrogen Peroxide Assay Kit was used to measure the H_2_O_2_ level, according to the manufacturer’s instructions. Firstly, the standard curve of the H_2_O_2_ dose-response was prepared. The H_2_O_2_ concentration for the samples was determined from the curve. Positive control wells (cells treated with 10 mM H_2_O_2_) and negative control wells (cells only and medium only) were used.

### 3.9. Mitochondrial Membrane Potential (MMP)

To measure mitochondrial membrane potential (MMP), Caco-2 cells (in the amount of 10,000 per well) were seeded in a 96-well black plate in complete culture medium. The cells were exposed to pulcherrimin suspensions (0.03; 0.1; 0.4; 1.6 mg/mL), as described in Section 3.5. The negative control contained cells in DMEM. The positive control contained cells in DMEM with CCCP (50 µM). The test samples were aspirated, and JC-1 (1 µg/mL) dye in DMEM (without FBS) was added to each well. The samples were incubated for 20 min (37 °C, 5% CO_2_). The fluorescence was measured (λ_ex_ 490 nm and λ_em_ 530 nm). For microscopic observations, Caco-2 cells were cultured in 8-well IBIDI LabTek II CC2 chambered coverslips with 50,000/well. The intracellular fluorescence of the cells was observed under a fluorescence microscope with 10× and 20× objectives.

### 3.10. Colony Formation Assay

For the colony formation assay, 200,000 Caco-2 cells were seeded into each well of a 6-well plate and cultured to reach 80% confluence. Next, the cells were washed with PBS and exposed to selected concentrations of pulcherrimin (0.03, 0.1, 0.4, and 1.6 mg/mL) for 60 min. The positive control was 200 mM H_2_O_2_. All cells in each well were harvested and counted in a haemocytometer. Next, 1000 cells were inoculated in each well of the 6-well plate and cultured for 7 days to enable the formation of colonies. The colonies were fixed with 3.7% paraformaldehyde for 15 min, air-dried, and stained with 0.1% crystal violet. The morphology of the Caco-2 cell monolayer was observed under a 10× objective in the abovementioned inverted microscope.

### 3.11. Adhesion Assay

For the adhesion assay, 10,000 Caco-2 cells were seeded in each well of a 96-well transparent plate in a complete culture medium. The cells were exposed to selected concentrations of pulcherrimin (0.03, 0.1, 0.4, and 1.6 mg/mL), as described in Section 3.5. Then, the test samples were aspirated, fixed with 3.7% paraformaldehyde (15 min), stained with 0.4% crystal violet (10 min), washed with PBS, dried, and observed under a 10× objective using an inverted microscope. In the next step, 70% ethanol was added to each well, and absorbance was measured at 570 nm in a microplate reader (reference 405 nm). The absorbance of untreated cells represented 100% viability. Cell adhesion (%) was calculated as follows: (sample OD/control OD) × 100%. The results were presented as the mean ± standard deviation (SD).

### 3.12. Cell Staining with DAPI and AO/PI

Nuclear changes in the Caco-2 cells after 24 h exposure to the selected concentration of pulcherrimin (1.6 mg/mL) were observed using 8-well IBIDI LabTek II CC2 chambered coverslips and DAPI staining (1 µg/mL) in the dark. For AO/PI staining, after 24 h of exposure to 0.1 and 1.6 mg/mL of pulcherrimin, the cells in the wells of a 6-well plate were detached from the plate, centrifuged (182× *g*, 5 min), decanted, and the pellet was stained with an AO (100 µg/mL) and PI (100 µg/mL) mixture (1:1, *v/v*). The morphology of the cells was observed for 15 min using a fluorescence microscope with a 20× objective.

### 3.13. Statistical Analysis

The results were subjected to a statistical analysis. The results are presented as the mean of three/four repeats ± standard deviation (SD). To analyse the inhibition of *Candida* and related-*Candida* strains growth by the *M. pulcherrima* clade, non-parametric tests were used for statistical analyses of the analysed parameters that did not follow a normal distribution (Shapiro-Wilk test). Differences regarding the analysed parameters were tested using the Kruskal-Wallis test (KW test), followed by a multiple comparison test (MCT) to indicate significant differences between the groups. In order to analyse the effect of the pulcherrimin production level on the inhibition properties, a One-way ANOVA was performed. The Spearman’s rank correlation coefficient (Spearman ρ) was performed to analyse the correlations between the pulcherrimin secretion and the growth inhibition zone diameter. A *p*-value < 0.05 was considered statistically significant. The KW, MCT, One-way ANOVA, and Independent sample *t*-Tests were performed using Statistica ver. 13.1 (StatSoft, Tulsa, OK, USA). The experiments on cell lines comprised the average values and a one-way ANOVA followed by Tukey’s multiple-comparisons post hoc test performed using OriginPro 6.1 software (OriginLab Corporation, Northampton, MA, USA) at a significance level of *p* ≤ 0.05.

## 4. Conclusions

Normally a benign commensal colonizer of mucosal surfaces, *Candida* spp. are also one of the most common fungal pathogens of humans, responsible for various invasive infections. We investigated the effect of the pulcherrimin producing *M. pulcherrima* clade on *Candida* and related *Candida* strains of different origins. Living *Metschnikowia* spp. cells repressed *Candida* and non-*Candida* growth, but environmental factors, including higher temperatures and the presence of silicate or peptones, had an inhibitory effect on the antifungal activity of *Metschnikowia* strains. Studies on the biological activity of pulcherrimin towards cell lines indicated that the red metabolite of *M. pulcherrima* has cytotoxic and antiproliferative activity, causing oxidative stress in cells that leads to apoptosis. This activity depends on the concentration of pulcherrimin. The damaging activity of pulcherrimin is probably connected with physical lesions caused by its particles as they adhere to outer cell membranes.

## Figures and Tables

**Table 1:** IC_50_ values of pulcherrimin determined for five cell lines evaluated on the basis of cytotoxicity curves.

Cell Line	IC_50_ [mg/mL]	Sequence of the Cytotoxicity
A-549	not detected	5—the weakest
Caco-2	0.36	1—the strongest
HeLa	1.16	3
HepG2	1.27	4
IEC-6	0.67	2

**Table 2:** Genotoxicity of pulcherrimin to Caco-2 human intestinal cells as % of DNA in the comet tail in the alkaline comet assay. Results presented as the mean for 50 cells analysed with each concentration of pulcherrimin ± S.E.M. Results were not significantly different, *p* ≤ 0.05.

Concentration [mg/mL]	DNA Damage [%] ± S.E.M.
0.01	4.84 ± 4.60
0.03	8.06 ± 2.15
0.1	6.33 ± 1.15
0.4	7.34 ± 1.33
1.6	7.09 ± 1.49

**Table 3:** Reactive oxidative species (ROS) generation in Caco-2 cells after 48-h exposure to pulcherrimin. Each data point represents the mean ± SD (*n* = 4). * Results significantly different from unexposed cells, *p* ≤ 0.05.

Concentration of Pulcherrimin [mg/mL]	Average Fluorescence Intensity [%] ± SD
Negative control (unexposed cells)	100 ± 3.9
0.03	109 ± 2.6
0.1	114 ± 2.5
0.4	125 ± 2.5 *
1.6	222 ± 3.4 *
200 mM H_2_O_2_ (positive control)	362 ± 3.9 *

**Table 4:** Hydrogen peroxide release in alive Caco-2 cells after 48-h exposure to pulcherrimin. Each data point represents the mean ± SD (*n* = 4). * Results significantly different from unexposed cells, *p* ≤ 0.05.

Concentration of Pulcherrimin [mg/mL]	H_2_O_2_ Production [µM] ± SD
Negative control (unexposed cells)	1.2 ± 1.9
0.03	6.5 ± 2.3 *
0.1	7.5 ± 1.8 *
0.4	7.5 ± 2.1 *
1.6	7.2 ± 2.3 *
10 mM H_2_O_2_ (positive control)	21.5 ± 2.9 *

**Table 5:** Mitochondrial membrane potential (MMP) depletion in Caco-2 cells after 48-h exposure to pulcherrimin. Each data point represents the mean ± SD (*n* = 4). * Results significantly different from unexposed cells, *p* ≤ 0.05.

Concentration of Pulcherrimin [mg/mL]	Mitochondrial Membrane Potential [%] ± SD
Negative control (unexposed cells)	100 ± 2.3
0.03	88 ± 2.3 *
0.1	83 ± 2.1 *
0.4	77 ± 2.2 *
1.6	59 ± 1.9 *
CCCP (positive control, 50 µM)	43 ± 1.9 *

**Table 6:** Yeast strains used in the study.

No	*Metschnikowia* Clade	Symbol	Origin
1	*M. sinensis* D1	LOCK 1135	apple fruits, Poland
2	*M. andauensis* D2	LOCK 1136	apple fruits, Poland
3	*M. sinensis* D3	LOCK 1137	raspberry fruits, Poland
4	*M. andauensis* D4	LOCK 1138	raspberry fruits, Poland
5	*M. andauensis* D5	LOCK 1139	grape berries, Poland
6	*M. andauensis* D6	LOCK 1140	grape berries, Poland
7	*M. andauensis* D7	LOCK 1141	red currant berries, Poland
8	*M. andauensis* D8	LOCK 1142	red currant berries, Poland
9	*M. sinensis* D9	LOCK 1143	strawberry fruits, Poland
10	*M. sinensis* D10	LOCK 1144	strawberry flowers, Poland
11	*M. pulcherrima* CCY145	CCY 29-2-145	grape berries, Slovakia
12	*M. pulcherrima* CCY149	CCY 29-2-149	grape berries, Slovakia
**No**	***Candida*** and non-*Candida* strains *	**Symbol**	**Origin**
1 *	*C. albicans*	CCY 29-3-163	blood, Slovakia
2	*C. albicans*	CCY 29-3-164	tonsils, Slovakia (resistant to antimycotics)
3	*C. albicans*	CCY 29-7-065	plum fruits, Slovakia
4	*C. glabrata*	CCY 26-20-3	mushroom, fruiting body, Czech Republic
5	*C. glabrata*	CCY 26-20-30	sputum, Slovakia (resistant to antimycotics)
6	*C. glabrata*	CCY 26-20-31	urine, Slovakia
7	*Meyerozyma guilliermondii* (syn. *Candida guilliermondii*)	CCY 29-4-38	sputum, unknown
8	*Meyerozyma guilliermondii* (syn. *Candida guilliermondii*)	CCY 29-4-39	peach tree, blossom, Slovakia
9	*Meyerozyma guilliermondii* (syn. *Candida guilliermondii*)	CCY 39-23-6	apple tree, leaves, Slovakia
10	*Pichia kudriavzevii* (syn. *Candida krusei*)	CCY 29-9-47	mouth, Slovakia (resistant to antimycotics)
11	*Pichia kudriavzevii* (syn. *Candida krusei*)	CCY 29-9-49	urine, Slovakia
12	*Pichia kudriavzevii* (syn. *Candida krusei*)	CCY 29-9-50	soil adjacent to apricot tree, Slovakia
13	*C. tropicalis*	CCY 29-7-64	plum tree, blossom, Slovakia
14	*C. tropicalis*	CCY 29-7-66	urine, Slovakia (resistant to antimycotics)
15	*C. tropicalis*	CCY 29-7-68	mouth, Slovakia
16	*C. parapsilosis*	CCY 29-20-29	apricot tree, blossom, Slovakia
17	*C. parapsilosis*	CCY 29-20-31	tongue, Slovakia
18	*C. parapsilosis*	CCY 29-20-32	trachea, Slovakia (resistant to antimycotics)
19	*Filobasidiella neoformans* var. *bacillispora* (syn. *Cryptococcus neoformans*)	CCY 17-1-8	clinical strain, Japan
20	*Filobasidiella neoformans* var. *bacillispora* (syn. *Cryptococcus neoformans*)	CCY 17-1-10	clinical strain, Japan

*—The gray colour denotes clinical strains.

**Table 7:** Agar media used to determine antifungal activity.

Methylene Blue Agar Variants	Symbol	Composition
YED-methylene blue agar	YED-MB	0.3% yeast extract (Merck), 0.5%glucose (Merck), 2% agar (Merck), 0.002% methylene blue (Merck), pH = 4.5
YEPD-methylene blue agar	YEPD-MB	0.3% yeast extract, 0.5%glucose, 1% peptone, 2% agar, 0.002% methylene blue, pH = 4.5
YEDSi-methylene blue agar	YEDSi-MB	0.3% yeast extract, 0.5%glucose, 2% agar, 0.1% sodium silicate (Lachema), 0.002% methylene blue, pH = 4.5

## Data Availability

Data are available upon request to the corresponding author.

## Supplementary Materials

The following supporting information can be downloaded at: https://www.mdpi.com/article/10.3390/molecules28135064/s1; Table S1: The pulcherrimin production by the *M. pulcherrima* clade. Statistically significant differences (KW test, followed by MCT) in pulcherrimin production are indicated in bold and letters (^A,B,C^), the *p* value is given below data for each strain; Table S2: The correlation analysis (Spearman ρ) of the pulcherrimin formation and the growth inhibition zone diameter; Table S3: The inhibition of clinical strains by the *M. pulcherrima* clade. Statistically significant differences in yeast inhibition by the strains belonging to the *M. pulcherrima* clade are indicated in bold and letters (^A,B^); the *p* value is given below data for each growth conditions; Table S4: The inhibition of non-clinical strains by the *M. pulcherrima* clade. Statistically significant differences in yeast inhibition by the strains belonging to the *M. pulcherrima* clade are indicated in bold and letters (^A,B^); the *p* value is given below data for each growth conditions; Table S5: Cytotoxic activity of pulcherrimin determined by PrestoBlue assay after 48 h exposition of cells. Each data point represents the mean ± SD, *n* ≥ 4. * Results significantly different from untreated cells (negative control), *p* ≤ 0.05.

## Sample Availability

Samples of the compounds and strains are not available from the authors.
