# Supplementary material for: The Metschnikowia pulcherrima Clade as a Model for Assessing Inhibition of Candida spp. and the Toxicity of Its Metabolite, Pulcherrimin
Source: Molecules. 2023 Jun 28;28(13):5064. doi: 10.3390/molecules28135064 (PMC10343249; doi:10.3390/molecules28135064)
Supplement: Supplementary file 1 [file molecules-28-05064-s001.zip › molecules-2449120-supplementary.pdf]

The *Metschnikowia pulcherrima* Clade as a Model for Assessing Inhibition of *Candida* spp. and the Toxicity of Its Metabolite, Pulcherrimin

Dorota Kregiel [1,2,*], Karolina H. Czarnecka-Chrebelska [3], Hana Schusterová [2], Renáta Vadkertiová [2] and Adriana Nowak [1]

[1] Department of Environmental Biotechnology, Faculty of Biotechnology and Food Sciences, Lodz University of Technology, Wolczanska 171/173, 90-530 Lodz, Poland
[2] Culture Collection of Yeasts, Institute of Chemistry, Slovak Academy of Sciences, Dúbravská Cesta 9, 845 38 Bratislava, Slovakia
[3] Department of Biomedicine and Genetics, Medical University of Lodz, Mazowiecka 5, 92-215 Lodz, Poland
* Correspondence: dorota.kregiel@p.lodz.pl

Supplementary

Table S1. The pulcherrimin production by the *M. pulcherrima* clade. Statistically significant differences (KW test, followed by MCT) in pulcherrimin production are indicated in bold and letters (^{A,B,C}), the p value is given below data for each strain.

Strain of the *M. pulcherrima* clade	Mean ± SD
CCY 145	142.000 ± 2.646
D1	48.000 ± 3.606
D2	64.667 ± 1.528
D3	20.333 ± 1.528
D4	**10.333 ± 1.528 ^{A,B}**
D5	81.667 ± 2.082
D6	**18.000 ± 1.000 ^{C}**
D7	70.000 ± 1.732
D8	25.000 ± 2.000
D9	**185.000 ± 4.583 ^{A,C}**
D10	**179.333 ± 1.528 ^{B}**
Statistically significant differences	^{A} p=0.009;<br>^{B} p=0.029;<br>^{C} p=0.043

Table S2. The correlation analysis (Spearman ϱ) of the pulcherrimin formation and the growth inhibition zone diameter.

Tested yeast strain	Spearman R	Correlation	p value (Spearman ϱ)
C. albicans CCY 29-3-163	0.553	positive	**0.033**
C. albicans CCY 29-3-164	0.553	positive	**0.033**
C. glabrata CCY 26-20-30	0.730	strong positive	**0.002**
C. glabrata CCY 26-20-31	0.841	strong positive	**0.000**
C. tropicalis CCY 29-7-66	0.833	strong positive	**0.000**
C. tropicalis CCY 29-7-68	0.711	strong positive	**0.003**
C. parapsilosis CCY 29-20-31	0.598	positive	**0.019**
C. parapsilosis CCY 29-20-32	0.693	strong positive	**0.004**
P. kudriavzevii CCY 29-9-47	0.868	strong positive	**0.000**
P. kudriavzevii CCY 29-9-49	0.680	strong positive	**0.005**
F. neoformans CCY 17-1-8	0.841	strong positive	**0.000**
F. neoformans CCY 17-1-10	0.863	strong positive	**0.000**
M. guillermondii CCY 29-4-38	0.830	strong positive	**0.000**
C. albicans CCY 29-7-65	0.678	strong positive	**0.005**
C. glabrata CCY 26-20-3	0.653	strong positive	**0.008**
C. tropicalis CCY 29-7-64	0.233	-	0.403
C. parapsilosis CCY 29-20-29	0.537	positive	**0.039**
P. kudriavzevii CCY 29-9-50	0.636	positive	**0.011**
M. guillermondii CCY 29-4-39	0.790	strong positive	**0.000**
M. guillermondii CCY 39-23-6	0.836	strong positive	**0.000**
Clinical strains	0.847	strong positive	**0.000**
Non-clinical strains	0.676	positive	**0.006**
**All strains**	**0.750**	**strong positive**	**0.001**

*) gray color denotes clinical strains

Table S3. The inhibition of clinical strains by the *M. pulcherrima* clade. Statistically significant differences in yeast inhibition by the strains belonging to the *M. pulcherrima* clade are indicated in bold and letters ([A,B]); the p value is given below data for each growth conditions.

Growth conditions	Tested clinical strain	***M. pulcherrima* clade**					
		**D1**	**D2**	**D9**	**D10**	**CCY 145**	**CCY 149**
YED-MB agar 22°C	*C. albicans* CCY 29-3-163	1.400 ± 0.100	1.500 ± 0.100	**1.667 ± 0.058** [A]	2.200 ± 0.000	**1.167 ± 0.058** [A]	1.500 ± 0.000
	C. albicans CCY 29-3-164	1.800 ± 0.173	1.000 ± 0.000	2.400 ± 0.100	2.200 ± 0.000	1.800 ± 0.000	1.400 ± 0.000
	C. glabrata CCY 26-20-30	1.167 ± 0.058	1.000 ± 0.000	**1.667 ± 0.058** [B]	**1.333 ± 0.115** [A,B,C]	**1.167 ± 0.058** [B]	1.400 ± 0.000
	C. glabrata CCY 26-20-31	1.333 ± 0.058	1.000 ± 0.000	2.200 ± 0.100	2.000 ± 0.100	1.600 ± 0.000	1.400 ± 0.000
	C. tropicalis CCY 29-7-66	1.140 ± 0.036	1.000 ± 0.000	2.000 ± 0.000	1.500 ± 0.000	1.500 ± 0.000	1.500 ± 0.000
	C. tropicalis CCY 29-7-68	1.800 ± 0.100	1.000 ± 0.000	2.600 ± 0.100	2.000 ± 0.000	1.667 ± 0.058	1.333 ± 0.058
	C. parapsilosis CCY 29-20-31	1.400 ± 0.100	1.000 ± 0.000	2.000 ± 0.000	**2.400 ± 0.000** [A]	**1.167 ± 0.058** [C]	**1.200 ± 0.000** [A]
	C. parapsilosis CCY 29-20-32	1.167 ± 0.058	1.000 ± 0.000	**1.600 ± 0.000** [C, D]	1.667 ± 0.058	1.600 ± 0.000	**1.000 ± 0.000** [B,C]
	P. kudriavzevii CCY 29-9-47	1.293 ± 0.081	1.167 ± 0.058	2.500 ± 0.000	1.500 ± 0.000	1.467 ± 0.058	1.667 ± 0.058
	P. kudriavzevii CCY 29-9-49	1.500 ± 0.100	1.000 ± 0.000	**3.000 ± 0.000** [A,B,C]	2.000 ± 0.000	1.333 ± 0.058	1.600 ± 0.000
	F. neoformans CCY 17-1-8	1.767 ± 0.153	1.000 ± 0.000	**2.800 ± 0.000** [D]	**2.333 ± 0.058** [B]	1.867 ± 0.058	**2.000 ± 0.000** [A,B]
	F. neoformans CCY 17-1-10	1.667 ± 0.058	1.333 ± 0.115	2.333 ± 0.058	**2.333 ± 0.058** [C]	1.833 ± 0.058	1.500 ± 0.000
	M. guillermondii CCY 29-4-38	1.500 ± 0.000	1.000 ± 0.000	2.167 ± 0.058	2.000 ± 0.000	**2.000 ± 0.000** [A,B,C]	**1.800 ± 0.000** [C]
				[A] p=0.045; [B] p=0.045; [C] p=0.013; [D] p=0.045	[A] p=0.013; [B] p=0.045; [C] p=0.045;	[A] p=0.031; [B] p=0.031; [C] p=0.031	[A] p=0.031; [B] p=0.008; [C] p=0.031
YED-MB agar 28°C	*C. albicans* CCY 29-3-163	**1.167 ± 0.058** [A]	**1.000 ± 0.000** [A]	**1.333 ± 0.058** [A,B]	1.400 ± 0.000	**1.000 ± 0.000** [A,B]	1.000 ± 0.000
	C. albicans CCY 29-3-164	**1.167 ± 0.058** [B]	1.167 ± 0.058	**2.000 ± 0.000** [A]	1.500 ± 0.000	1.500 ± 0.000	1.000 ± 0.000
	C. glabrata CCY 26-20-30	**1.167 ± 0.058** [C]	**1.000 ± 0.000** [B]	**1.267 ± 0.058** [C]	**1.167 ± 0.058** [A,B]	1.183 ± 0.076	1.167 ± 0.058
	C. glabrata CCY 26-20-31	1.360 ± 0.036	**1.000 ± 0.000** [C]	1.550 ± 0.050	**1.167 ± 0.058** [C,D]	1.183 ± 0.076	1.200 ± 0.000
	C. tropicalis CCY 29-7-66	1.500 ± 0.000	1.400 ± 0.000	1.667 ± 0.058	1.333 ± 0.058	1.500 ± 0.000	1.000 ± 0.000
	C. tropicalis CCY 29-7-68	1.333 ± 0.058	1.167 ± 0.058	1.500 ± 0.000	1.400 ± 0.000	1.433 ± 0.058	1.083 ± 0.029
	C. parapsilosis CCY 29-20-31	1.333 ± 0.058	1.150 ± 0.050	**2.100 ± 0.000** [B,C]	**1.600 ± 0.000** [A,C]	1.333 ± 0.058	1.083 ± 0.029

	C. parapsilosis CCY 29-20-32	1.417 ± 0.029	1.333 ± 0.058	1.667 ± 0.058	1.500 ± 0.000	1.550 ± 0.050	1.360 ± 0.036
	P. kudriavzevii CCY 29-9-47	**1.617 ± 0.029** A,B,C	**1.500 ± 0.000** A,B,C	1.667 ± 0.058	1.500 ± 0.000	1.613 ± 0.023	1.333 ± 0.058
	P. kudriavzevii CCY 29-9-49	1.500 ± 0.100	1.333 ± 0.058	1.733 ± 0.058	**1.600 ± 0.000** B,D	1.310 ± 0.036	1.000 ± 0.000
	F. neoformans CCY 17-1-8	1.333 ± 0.058	1.167 ± 0.058	1.550 ± 0.050	1.333 ± 0.058	1.500 ± 0.000	1.167 ± 0.058
	F. neoformans CCY 17-1-10	1.500 ± 0.000	1.333 ± 0.115	1.500 ± 0.000	1.333 ± 0.058	**1.667 ± 0.058** A	1.167 ± 0.058
	M. guillermondii CCY 29-4-38	1.433 ± 0.058	1.333 ± 0.115	1.667 ± 0.058	1.550 ± 0.050	**1.623 ± 0.025** B	1.333 ± 0.058
		A p=0.035; B p=0.035; C p=0.035	A p=0.031; B p=0.031; C p=0.031	A p=0.040; B p=0.023; C p=0.011;	A p=0.037; B p=0.037; C p=0.037; D p=0.037	A p=0.017; B p=0.035;	
YEPD-MB agar 22°C	*C. albicans* CCY 29-3-163	1.287 ± 0.032	1.000 ± 0.000	**1.293 ± 0.012** A,B	**1.333 ± 0.058** A,B	**1.000 ± 0.000** A	1.000 ± 0.000
	C. albicans CCY 29-3-164	1.250 ± 0.000	1.000 ± 0.000	2.000 ± 0.000	2.000 ± 0.000	**1.000 ± 0.000** B	1.000 ± 0.000
	C. glabrata CCY 26-20-30	1.433 ± 0.058	1.000 ± 0.000	1.850 ± 0.050	1.710 ± 0.017	1.283 ± 0.029	1.142 ± 0.052
	C. glabrata CCY 26-20-31	**1.000 ± 0.000** A,B,	1.000 ± 0.000	1.833 ± 0.058	1.703 ± 0.006	**1.000 ± 0.000** C	1.000 ± 0.000
	C. tropicalis CCY 29-7-66	1.367 ± 0.058	1.000 ± 0.000	**2.467 ± 0.058** B	2.167 ± 0.029	1.750 ± 0.050	1.000 ± 0.000
	C. tropicalis CCY 29-7-68	1.223 ± 0.025	1.000 ± 0.000	2.137 ± 0.015	1.857 ± 0.006	1.567 ± 0.058	1.133 ± 0.058
	C. parapsilosis CCY 29-20-31	**1.567 ± 0.058** A	1.133 ± 0.058	**1.500 ± 0.000** C	1.667 ± 0.029	1.433 ± 0.058	1.000 ± 0.000
	C. parapsilosis CCY 29-20-32	1.217 ± 0.015	1.000 ± 0.000	2.140 ± 0.017	1.850 ± 0.000	1.750 ± 0.000	1.000 ± 0.000
	P. kudriavzevii CCY 29-9-47	1.383 ± 0.029	1.000 ± 0.000	**2.600 ± 0.000** A,C	**2.300 ± 0.000** A	**1.853 ± 0.006** A,B,C	1.240 ± 0.010
	P. kudriavzevii CCY 29-9-49	**1.500 ± 0.100** B,	1.117 ± 0.029	2.000 ± 0.000	**2.333 ± 0.058** B,C	1.383 ± 0.029	1.000 ± 0.000
	F. neoformans CCY 17-1-8	1.325 ± 0.106	1.000 ± 0.000	1.850 ± 0.050	1.850 ± 0.050	1.290 ± 0.017	1.240 ± 0.017
	F. neoformans CCY 17-1-10	1.293 ± 0.012	1.000 ± 0.000	1.667 ± 0.029	**1.500 ± 0.000** C	1.280 ± 0.017	1.000 ± 0.000
	M. guillermondii CCY 29-4-38	1.167 ± 0.058	1.000 ± 0.000	1.833 ± 0.058	1.833 ± 0.058	1.707 ± 0.012	1.433 ± 0.058
		A p=0.013; B p=0.046;		A p=0.008; B p=0.031; C p=0.031;	A p=0.020; B p=0.013; C p=0.045;	A p=0.031; B p=0.031; C p=0.031	
YEDSi-MB agar 22°C	*C. albicans* CCY 29-3-163	1.000 ± 0.000	1.000 ± 0.000	1.000 ± 0.000	1.000 ± 0.000	1.000 ± 0.000	1.000 ± 0.000
	C. albicans CCY 29-3-164	1.250 ± 0.000	1.000 ± 0.000	1.140 ± 0.010	1.140 ± 0.017	1.000 ± 0.000	1.140 ± 0.017
	C. glabrata CCY 26-20-30	1.293 ± 0.012	1.000 ± 0.000	1.000 ± 0.000	1.000 ± 0.000	1.000 ± 0.000	1.000 ± 0.000

	C. glabrata CCY 26-20-31	1.000 ± 0.000	1.000 ± 0.000	1.000 ± 0.000	1.000 ± 0.000	1.000 ± 0.000	1.000 ± 0.000
	C. tropicalis CCY 29-7-66	1.000 ± 0.000	1.000 ± 0.000	1.000 ± 0.000	1.000 ± 0.000	1.000 ± 0.000	1.000 ± 0.000
	C. tropicalis CCY 29-7-68	1.133 ± 0.058	1.133 ± 0.058	1.140 ± 0.017	1.140 ± 0.017	1.000 ± 0.000	1.000 ± 0.000
	C. parapsilosis CCY 29-20-31	1.000 ± 0.000	1.000 ± 0.000	1.000 ± 0.000	1.000 ± 0.000	1.000 ± 0.000	1.000 ± 0.000
	C. parapsilosis CCY 29-20-32	1.133 ± 0.058	1.140 ± 0.017	1.140 ± 0.017	1.140 ± 0.017	1.000 ± 0.000	1.000 ± 0.000
	P. kudriavzevii CCY 29-9-47	1.000 ± 0.000	1.000 ± 0.000	1.000 ± 0.000	1.000 ± 0.000	1.000 ± 0.000	1.000 ± 0.000
	P. kudriavzevii CCY 29-9-49	1.633 ± 0.058	1.333 ± 0.058	1.567 ± 0.058	1.567 ± 0.058	1.857 ± 0.012	1.833 ± 0.058
	F. neoformans CCY 17-1-8	1.500 ± 0.000	1.000 ± 0.000	1.500 ± 0.000	1.500 ± 0.000	1.500 ± 0.000	1.000 ± 0.000
	F. neoformans CCY 17-1-10	1.500 ± 0.000	1.250 ± 0.000	1.860 ± 0.010	1.500 ± 0.000	1.250 ± 0.000	1.000 ± 0.000
	M. guillermondii CCY 29-4-38	1.000 ± 0.000	1.000 ± 0.000	1.000 ± 0.000	1.000 ± 0.000	1.000 ± 0.000	1.000 ± 0.000

Table S4. The inhibition of non-clinical strains by the *M. pulcherrima* clade. Statistically significant differences in yeast inhibition by the strains belonging to the *M. pulcherrima* clade are indicated in bold and letters ([A,B]); the p value is given below data for each growth conditions.

Growth conditions	Tested non-clinical strain	***M. pulcherrima* clade**					
		**D1**	**D2**	**D9**	**D10**	**CCY145**	**CCY149**
YED-MB agar 22°C	*C. albicans* CCY 29-7-65	1.667 ± 0.058	1.00 ± 0.00	**2.40 ± 0.00** [A]	2.00 ± 0.00	1.333 ± 0.058	**1.00 ± 0.00** [A]
	C. glabrata CCY 26-20-3	1.333 ± 0.058	1.00 ± 0.00	2.333 ± 0.058	2.00 ± 0.00	**1.00 ± 0.00** [A]	**2.00 ± 0.00** [A,B]
	C. tropicalis CCY 29-7-64	1.333 ± 0.058	1.167 ± 0.058	1.567 ± 0.058	1.20 ± 0.00	1.167 ± 0.029	1.167 ± 0.029
	C. parapsilosis CCY 29-20-29	2.00 ± 0.00	1.00 ± 0.00	2.20 ± 0.00	2.00 ± 0.00	**1.80 ± 0.00** [A,B]	1.60 ± 0.00
	P. kudriavzevii CCY 29-9-50	1.333 ± 0.058	1.00 ± 0.00	1.80 ± 0.00	1.567 ± 0.058	**1.00 ± 0.00** [B]	**1.00 ± 0.00** [B]
	M. guillermondii CCY 29-4-39	1.333 ± 0.058	1.167 ± 0.058	2.00 ± 0.00	1.617 ± 0.029	1.667 ± 0.058	1.40 ± 0.00
	M. guillermondii CCY 39-23-6	1.333 ± 0.058	1.00 ± 0.00	**1.50 ± 0.00** [A]	1.50 ± 0.00	1.40 ± 0.00	1.40 ± 0.00
				[A] p= 0.016		[A] p=0.0237; [B] p=0.0237;	[A] p=0.0237; [B] p=0.0237
YED-MB agar 28°C	*C. albicans* CCY 29-7-65	1.333 ± 0.058	1.000 ± 0.000	1.567 ± 0.058	1.183 ± 0.029	1.463 ± 0.032	1.000 ± 0.000
	C. glabrata CCY 26-20-3	1.333 ± 0.058	1.000 ± 0.000	1.500 ± 0.000	1.360 ± 0.036	1.333 ± 0.058	1.177 ± 0.025
	C. tropicalis CCY 29-7-64	**1.183 ± 0.029** [A]	1.000 ± 0.000	**1.333 ± 0.058** [A]	**1.177 ± 0.025** [A]	**1.083 ± 0.076** [A]	1.000 ± 0.000
	C. parapsilosis CCY 29-20-29	1.500 ± 0.000[A]	1.167 ± 0.029	1.833 ± 0.058	1.500 ± 0.000	1.333 ± 0.058	1.000 ± 0.000
	P. kudriavzevii CCY 29-9-50	1.500 ± 0.000	1.333 ± 0.058	1.667 ± 0.058	1.500 ± 0.000	1.333 ± 0.058	1.750 ± 0.050
	M. guillermondii CCY 29-4-39	1.500 ± 0.000	1.000 ± 0.000	1.600 ± 0.000	1.333 ± 0.058	1.500 ± 0.000	1.090 ± 0.036
	M. guillermondii CCY 39-23-6	**1.567 ± 0.058** [A]	1.000 ± 0.000	**2.000 ± 0.000** [A]	**1.800 ± 0.000** [A]	**1.667 ± 0.058** [A]	1.083 ± 0.076
		[A] p= 0.024		[A] p= 0.008	[A] p=0.018	[A] p=0.008	
YEPD-MB agar 22°C	*C. albicans* CCY 29-7-65	1.133 ± 0.058	1.000 ± 0.000	1.873 ± 0.025	1.873 ± 0.025	1.000 ± 0.000	1.000 ± 0.000
	C. glabrata CCY 26-20-3	1.400 ± 0.000	1.000 ± 0.000	2.333 ± 0.058	2.167 ± 0.058	1.000 ± 0.000	1.000 ± 0.000
	C. tropicalis CCY 29-7-64	1.333 ± 0.058	1.000 ± 0.000	1.667 ± 0.058	1.667 ± 0.058	1.000 ± 0.000	1.000 ± 0.000
	C. parapsilosis CCY 29-20-29	**1.883 ± 0.029** [A]	1.000 ± 0.000	2.200 ± 0.100	1.833 ± 0.058	1.000 ± 0.000	1.000 ± 0.000
	P. kudriavzevii CCY 29-9-50	**1.000 ± 0.000** [A,B]	1.000 ± 0.000	**1.167 ± 0.058** [A]	**1.167 ± 0.058** [A]	1.000 ± 0.000	1.000 ± 0.000
	M. guillermondii CCY 29-4-39	1.433 ± 0.058	1.000 ± 0.000	**2.500 ± 0.000** [A]	**2.000 ± 0.000** [A]	1.433 ± 0.058	1.393 ± 0.021
	M. guillermondii CCY 39-23-6	**1.833 ± 0.058** [B]	1.000 ± 0.000	1.833 ± 0.058	1.667 ± 0.058	1.500 ± 0.000	1.333 ± 0.058

		^{A} p= 0.013; ^{B} p=0.041		^{A} p= 0.008	^{A} p=0.064		
YEDSi-MB agar 22°C	*C. albicans* CCY 29-7-65	**1.000 ± 0.000** $^{\mathbf{A}}$	1.000 ± 0.000	1.000 ± 0.000	1.000 ± 0.000	1.000 ± 0.000	1.000 ± 0.000
	C. glabrata CCY 26-20-3	1.250 ± 0.050	1.000 ± 0.000	1.000 ± 0.000	1.000 ± 0.000	1.000 ± 0.000	1.000 ± 0.000
	C. tropicalis CCY 29-7-64	1.250 ± 0.050	1.000 ± 0.000	1.400 ± 0.000	1.333 ± 0.058	1.000 ± 0.000	1.000 ± 0.000
	C. parapsilosis CCY 29-20-29	**1.000 ± 0.000** $^{\mathbf{B}}$	1.000 ± 0.000	1.000 ± 0.000	1.000 ± 0.000	1.000 ± 0.000	1.000 ± 0.000
	P. kudriavzevii CCY 29-9-50	1.500 ± 0.000	1.143 ± 0.012	1.333 ± 0.058	1.143 ± 0.012	1.000 ± 0.000	1.000 ± 0.000
	M. guillermondii CCY 29-4-39	1.143 ± 0.012	1.133 ± 0.006	1.333 ± 0.058	1.333 ± 0.058	1.293 ± 0.012	1.000 ± 0.000
	M. guillermondii CCY 39-23-6	**1.567 ± 0.058** $^{\mathbf{A,B}}$ ^{A} p= 0.033; ^{B} p=0.033	1.217 ± 0.015	1.500 ± 0.000	1.667 ± 0.058	1.633 ± 0.058	1.567 ± 0.058

Table S5. Cytotoxic activity of pulcherrimin determined by *PrestoBlue* assay after 48 h exposition of cells. Each data point represents the mean ± SD, $n \geq 4$. * Results significantly different from untreated cells (negative control), $p \leq 0.05$.

Concentration [mg/mL]	Cytotoxicity [%] ± SD				
	**A-549**	**Caco-2**	**HeLa**	**HepG2**	**IEC-6**
0.01	4.48 ± 11.89	7.44 ± 0.35	17.12 ± 0.95*	0.36 ± 0.96	5.98 ± 0.75
0.03	3.11 ± 3.67	10.96 ± 0.63*	10.04 ± 1.87	5.97 ± 1.52	9.41 ± 0.78*
0.05	6.45 ± 4.68	13.89 ± 5.84*	15.42 ± 4.66*	6.49 ± 6.17	17.87 ± 2.49*
0.10	4.21 ± 1.89	16.47 ± 6.02*	14.90 ± 2.78*	12.27 ± 6.81*	21.28 ± 2.76*
0.20	9.61 ± 2.64*	25.04 ± 5.47*	6.56 ± 6.23	-1.55 ± 3.60	25.53 ± 3.34*
0.40	14.09 ± 3.52*	55.42 ± 5.09*	24.55 ± 6.18*	8.53 ± 5.61	28.89 ± 3.16*
0.80	20.17 ± 1.64*	71.50 ± 4.08*	27.69 ± 7.18*	12.50 ± 4.82*	59.46 ± 3.28*
1.60	24.38 ± 3.64*	87.49 ± 5.73*	75.25 ± 10.11*	74.33 ± 6.65*	69.22 ± 2.54*
3.20	36.47 ± 2.35*	87.58 ± 10.05*	77.20 ± 6.43*	78.21 ± 8.26*	71.82 ± 0.31*
